# Supplementary material for: Pre-clinical evaluation of a low-cost tool for skin temperature measurements as a proxy to assess autonomic nerve function in leprosy neuropathy
Source: PLoS One. 2025 Jul 11;20(7):e0327815. doi: 10.1371/journal.pone.0327815 (PMC12250527; doi:10.1371/journal.pone.0327815)
Supplement: Attachment — Submitted filename: Reviewers comments and authors rebuttal v2.docx [file pone.0327815.s001.docx]

Dear editor,

We thank both reviewers for their efforts to review our manuscript thoroughly. Please find below our responses and the changes made to the manuscript to address the comments. We believe to have addressed the issues raised sufficiently. The input and effort from both reviewers has really helped to increase the quality and readability of the manuscript.

Regards

Arjan

| **Reviewer #1: General Comments** | **Author’s response and changes** |
| --- | --- |
| 1. The findings of this study show that a low-cost IR camera can be used to measure STR. However, these results alone do not substantiate the claim in the title that this low-cost tool can assess autonomic nerve function impairment. Although the future research in this direction is mentioned in the conclusion, the current study does not establish a direct relationship between skin temperature and autonomic nerve function impairment. Therefore, additional experiments specifically designed to explore this relationship should be incorporated into the main body of the paper. | Agreed. Therefore, we are currently doing a clinical research study where we enroll both patients and healthy subjects, to study the relation between skin temperature and autonomic nerve function impairment.  We have aimed to clarify this in our manuscript that skin temperature COULD be a proxy for ANF. Our claims and conclusions have been written as such. I see the issue in the title.  *Changed the title to underline this: Pre-clinical evaluation of a low-cost tool for skin temperature measurements as a proxy to assess autonomic nerve function impairment.* |
| 2. In the discussion section, it is mentioned that the observed differences among various sensors might not only stem from issues related to accuracy or precision, but also from actual physical differences in skin temperature across the three measurement locations. Employing a cross-sensor validation test could effectively address this issue | Agreed. But we did not include this in our study design, so at this stage this cannot be added to the manuscript.  *Added to the discussion: Further work, for instance performing a cross-sensor validation test could clarify this issue.* |
| **Reviewer 1: Specific Comments** |  |
| 1. In the manuscript, it is mentioned that the resulting STR curve should be recorded for 15 minutes. Why choose 15 minutes? Why not 10 minutes or 20 minutes? | This is based on literature; in healthy controls 50-70% recovery happened after 5 minutes, and after 15 minutes most healthy controls approach baseline temperature.  *Added rationale and reference to the manuscript.* |
| 2. Why use an IR video camera? What are the advantages of an IR video camera over contact temperature sensors? There are many low-cost portable contact temperature sensors could be chosen. | The main advantages of an IR video camera are that it provides high-resolution spatial temperature information across every image, allowing the researchers to change locations of ROI during the analysis, if needed, and to deploy other ways to analyze the videos, for instance, using machine learning/image processing techniques, etc. This would not be possible with contact sensors.  *In the requirements section added:* *This provided flexibility to add/change ROI during post-processing, and the option to deploy other image processing techniques if necessary* |
| 3. Why use a Python script to extract video to numeric data and use a MATLAB script for combining and processing the extracted data? Why use the same language? | This is the result of practicalities; the script for data extraction from a video was written by a person skilled in Python, and the script to combine the resulting data across many subjects was written at a later stage by someone skilled in Matlab.  *No change needed to the manuscript.* |
| 4. In the third paragraph of the abstract, the last sentence “both within as between…” is inappropriate. | I was aiming to say that all curves follow a similar trajectory (slow, passive recovery first, then followed by a steep, active recovery) but that the change from passive to active recovery varies within subjects (when repeating 5 times on 1 subject) and between subject (testing all included subjects 5 times).  *Changed wording to: Although the general shape of the STR was similar, the location of the steep increase in recovery varied strongly within and between subjects.* |
| 5. In the methods part, how to quantify the submersion depth of subjects’ hands to guarantee repeatability? | Immersion was instructed as ‘up till wrist’.  *Added: up to the wrists.* |
| 6. In the Camera selection part, the expression of ‘EUR 2000 – 18.000’ is incorrect. | *Removed this part as it was only to contrast our camera cost against higher-end cameras.* |
| 7. The expression of ‘warming phase’ of Figure 2 is not mentioned in other parts, does it mean ‘recovery phase’? | Apologies, this is remaining old wording.  *Changed to recovery phase.* |
| 8. The IR camera position in the left picture of Figure 3 is not clear, so I highly recommend using distinctive curves and arrows to indicate the position. | I can see that our picture is not optimal.  *Changed Figure 3 for a clearer picture that demonstrates the various parts of the setup, including more detailed pictures of the IR camera.* |
| 9. In the part of Camera accuracy of skin temperature measurement, how to guarantee the same temperature changes when drying the hand before placing the hand in the measurement setup among different repeated experiments? | Our interest here is not a reproducible initial condition, but to provide a cooled hand as initial state that then gradually warms up to provide a range of skin temperatures to the skin temperature sensor and to the IR video camera, and that these measurement methods can be compared across this range of temperatures.  *Added to this section: The intend of cooling the hand was not to reach a certain reproducible temperature, but to present a skin temperature range to the temperature sensors that is relevant for the clinical application, allowing skin temperature comparison between sensors across this range.* |
| 10. In the part of Camera accuracy of skin temperature measurement, it is mentioned that … until the hand temperature had stabilized. What is the criterion for hand temperature stabilization? | As above, our aim was not a reproducible end point of the test, but only to provide a range of skin temperatures to compare the temperature sensing methods.  *Changed sentence to: The average IR temperature and the temperature sensor values were recorded every 1 minute while the skin temperature was in the range between 26 and 36* °C*, or until the hand temperature no longer increased..* |
| 11. In the part of Camera accuracy of skin temperature measurement, the expression of ‘The left panel displays the Intra-Class Correlation plot between the two’ is not clear. | To enhance clarity the wording is changed to:  *…between the Mindray skin temperature sensor readings and the IR camera readings.* |
| 12. In the first paragraph of the discussion, the expression of ‘A Python script was made to extract the temperatures of 10 ROIs…’ is not in accordance with the 12 ROIs in other parts. | Apologies, this is a mistake, it should be 12 ROI like elsewhere.  *Corrected 10 to 12 ROI.* |
| 13. In the manuscript, it is mentioned that between observers, the largest differences were found for T0 and t25. Can script-based automated recognition replace manual work? | Yes, we are actually working on a script using image processing and machine learning techniques to identify the ROI in a video frame, only requiring the hands to be present, and not necessarily to be in a fixed location. This model was trained on RGB images, and still struggles with some low-contrast situations in the IR images that we are still trying to solve.  *Added*: *A potential approach to solve this issue could be the deployment of a machine learning model that identifies the ROI automatically.* |

| **Reviewer #2: General comments** | **Author’s response and changes** |
| --- | --- |
| This is a well-designed study. It's believed the proposed low-cost experimental setup can not only be used for assessment of peripheral autonomic nerve function but also for assessment of microcirculation function. The reviewer has only several small suggestions. | Thank you for your kind words. Indeed the methodology could be applied for other applications as well. |
| **Reviewer #2: Specific comments** |  |
| 1. The low-cost IR camera is the key instrument. One picture showing the camera and the connection to the mobile phone may be presented in Fig. 4 beside the fixing supporter and the mobile phone. | I can see that our picture is not optimal.  *Changed Figure 3 for a clearer picture that demonstrates the various parts of the setup, including more detailed pictures of the IR camera.*  Figure 4 is intended to show only the situation around this particular accuracy test, no change needed here. |
| 2. The designed fixing suporter is a very good apparatus for avoiding hand moving. But in some circumstances the temperatures of the supporter may be close to the finger temperature （for example, in Fig. 5 the boundary between the 3rd and 4th finger seems to be a little bit blurred）. The method to identify the finger boundary may be explained more in details. | Low contrast situations caused by minimal temperature differences between parts of the hand and the background can be challenging indeed. We minimized the risk by letting the script select a frame with high contrasts and it generally works well. Cases where the script presents a frame to select the ROI where it is really impossible to distinguish between hand and background are really rare.  In the particular case of Fig 5, here the finger is not properly placed in fixation mold. As we are measuring the temperature in the hand palm during this particular test this does not affect the outcomes.  *Added to the Python script section: The script selects a frame that has maximum difference in contrast between the mean value of the highest 20% and lowest 20% of pixels in the frame, and presents that frame to the user for ROI selection.* |
| 3. In the introduction part, it's mentioned that " various studies [17–22] have been done, mainly using high-end or not so portable infrared (IR) cameras, or contact temperature sensors". Here， more detailed descriptions may be given to show how the skin temperature variations are associated with ANF. Similarly, in discussion part, it's written that "other factors besides room temperature, time of the day, and acclimatization are influencing the outcomes". It's considered that skin temperature is closely related to skin blood flow and metabolic heat generation. Here, the discusion may be more extended | Expanded a little in the introduction to paint a clearer image without going into detail (which is not the goal of this introduction).  Expanded the discussion a little to indicate possible other factors influencing the STR curve. Without being complete or conclusive. We preserve that for our larger clinical study. |
